# Is the association between mothers’ autistic traits and childhood autistic traits moderated by maternal pre-pregnancy body mass index?

**DOI:** 10.1186/s13229-023-00578-x

**Published:** 2023-12-08

**Authors:** Novika Purnama Sari, Alexandros Tsompanidis, Rama J. Wahab, Romy Gaillard, Ezra Aydin, Rosemary Holt, Carrie Allison, Simon Baron-Cohen, Marinus H. van IJzendoorn, Pauline W. Jansen

**Affiliations:** 1 Department Psychology, Education & Child Studies, Erasmus University Rotterdam, Rotterdam, The Netherlands; 2 Department of Child & Adolescent Psychiatry/Psychology, Erasmus University Medical Centre, Rotterdam, The Netherlands; 3 Generation R Study Group, Erasmus University Medical Centre Rotterdam, Rotterdam, The Netherlands; 4 Research Department of Clinical, Educational and Health Psychology, University College London, London, UK; 5 Department of Psychiatry, Autism Research Centre, University of Cambridge, Cambridge, UK; 6 Vagelos College of Physicians and Surgeons, Columbia University, New York, USA; 7 Department of Psychology, University of Cambridge, Cambridge, UK; 8 Department Clinical and Developmental Neuropsychology, University of Groningen, Groningen, The Netherlands

**Keywords:** Autistic traits, Pre-pregnancy body mass index, Mothers, Children

## Abstract

**Background:**

Previous studies showed that there is a positive association between mothers’ and children’s autistic traits. We also tested if this association is more pronounced in mothers with a higher pre-pregnancy body mass index (BMI).

**Method:**

The study was embedded in two cohorts with information available for 4,659 participants from the Generation R and for 179 participants from the Cambridge Ultrasound Siblings and Parents Project (CUSP) cohort. In both cohorts, maternal autistic traits were assessed using the short form of the Autism Spectrum Quotient, and information about maternal height and weight before pregnancy was obtained by questionnaire. Child autistic traits were assessed with the short form of Social Responsiveness Scale in Generation R (*M* = 13.5 years) and with the Quantitative Checklist for Autism in Toddlers (Q-CHAT) in the CUSP cohort (*M* = 1.6 years).

**Result:**

Higher maternal autistic traits were associated with higher autistic traits in toddlerhood (CUSP cohort; *β*_*adjusted*_ = 0.20, *p* < 0.01), in early childhood (Generation R; *β*_*adjusted*_ = 0.19, *p* < 0.01), and in early adolescence (Generation R; *β*_*adjusted*_ = 0.16, *p* < 0.01). Furthermore, a higher maternal pre-pregnancy BMI was associated with higher child autistic traits, but only in Generation R (*β*_*adjusted*_ = 0.03, *p* < 0.01). There was no significant moderating effect of maternal pre-pregnancy BMI on the association between autistic traits of mothers and children, neither in Generation R nor in CUSP. In addition, child autistic traits scores were significantly higher in mothers who were underweight and in mothers who were overweight compared to mothers with a healthy weight.

**Conclusion:**

We confirm the association between maternal and child autistic traits in toddlerhood, early childhood, and early adolescence. Potential interacting neurobiological processes remain to be confirmed.

**Supplementary Information:**

The online version contains supplementary material available at 10.1186/s13229-023-00578-x.

## Introduction

Autism is a neurodevelopmental condition defined by difficulties in social communication and interaction, and the presence of stereotyped behaviours and interests (1). The aetiology is multifactorial with both genetic and environmental factors playing a role. Indeed, previous studies have shown that autistic traits are largely heritable (2–4), reflected in a positive relationship between autistic traits of children and their mothers (5). Many studies (6–9) have also explored the broad autism phenotype (BAP) within families impacted by autism, presenting supporting evidence for the transmission of autistic traits across generations. However, most studies focused on families with autism diagnoses and there has been relatively little research examining autistic traits of mothers and of children in larger samples or across the autism spectrum. Several studies (2, 10, 11) have emphasized the importance of studying autistic traits with a prospective cohort design in the general population, to increase numbers, and to include the full distribution of traits as part of a wider continuous spectrum in the population. Thereby, large study samples allow us to evaluate whether certain environmental characteristics partly explain the link between mothers’ and children’s autistic traits, to better understand the intergenerational traits’ transmission and to enable earlier diagnosis and intervention after birth.

Three meta-analyses indicate an association between maternal body mass index (BMI) and clinically diagnosed autism in the offspring (12–14). These studies revealed that higher maternal BMI increases the likelihood of autism in children. For every 5 kg/m^2^ increment in BMI, there is a 16% increase in autism likelihood. However, the opposite has also been found, as mothers deemed underweight also have an elevated likelihood for autism among offspring, particularly in older mothers (15). In summary, previous work showed that mothers with a high level of autistic traits or who are overweight or underweight (16) are more likely to have children with autism.

Maternal pre-pregnancy BMI, as an indicator of maternal health and nutritional status (17), could impact prenatal environment (e.g., endocrine homeostasis (18), hormone (19) and subsequently affect the developing foetus (20). In a study by Lyall et al., (2011), it was found that maternal pre-pregnancy BMI at age 18 was significantly associated with an increased likelihood of autism. Considering the multifactorial aetiology of autism (21) and previous indications of familial risk (gene)—environment interactions (22), these two risk factors (maternal autistic traits and weight) might also exacerbate each other. Therefore, in the current study, we examine the relationship between autistic traits in mothers and their children, and explore whether maternal pre-pregnancy BMI moderates this relationship.

For this, we use data from the Generation R and the Cambridge Ultrasound Siblings and Parents (CUSP) cohorts. Considering our sample comprises children from both the UK and the Netherlands, we acknowledge that there are different preferences regarding terminology within the autism community in these two countries (23, 24). To maintain consistency, we use person-first language throughout this paper.

## Methods

### Design and study participants

The study included two different cohorts: the Generation R and the Cambridge Ultrasound and Pregnancy (CUSP) cohort. Generation R is a large population-based cohort of mothers and children in Rotterdam, the Netherlands (25). Pregnant women were enrolled between 2002 and 2006 (participation rate: 61%) and, together with their children, have been followed longitudinally to the present day. Approval for the study was obtained from Medical Ethical Committee of the Erasmus Medical Centre. Consent for postnatal participation was available for 6,706 children and mothers. Participants with missing data on child autistic traits (*N* = 2,047) were excluded, leaving 4,659 participants for the analyses.

**Fig. 1:**
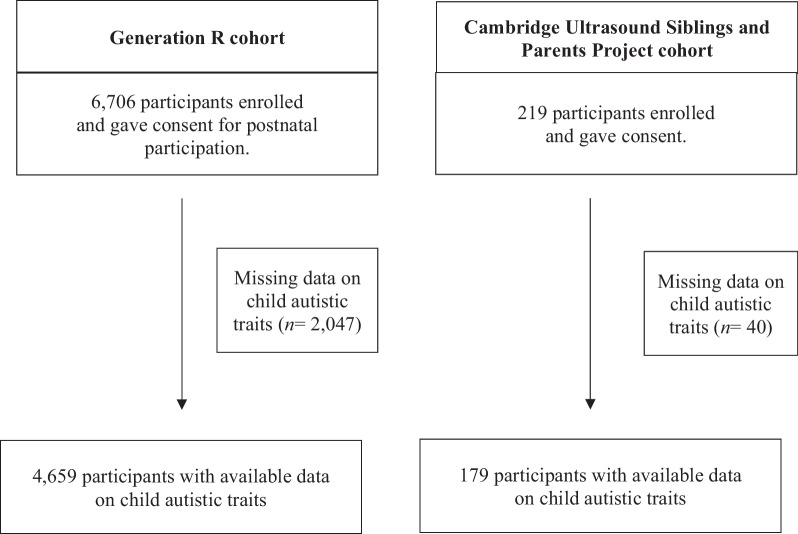
Flow chart of participants included for analyses

The Cambridge Ultrasound and Pregnancy (CUSP) cohort recruited mothers before or during their routine 20-week ultrasound scan, between 2016 and 2018 at the Rosie Hospital, Cambridge University Hospitals NHS Foundation Trust. Participating mothers gave informed consent for access to all their pregnancy-related clinical records, test results and the biological samples obtained during routine clinical care. A favourable ethical opinion was given by the East of England Cambridge Central Research Ethics Committee (REC Ref 16/EE/0004) and the Research and Development Department of Cambridge University Hospitals. Eligibility inclusion criteria for the CUSP cohort were as follows: (1) little/no consumption of alcohol during pregnancy, (2) no smoking or recreational drug use during pregnancy, (3) a singleton pregnancy, and (4) measurements indicated no intrauterine growth restrictions or large-for-gestational age foetuses (26, 27). Consent to use maternal and child data was available for 219 children. Participants with missing data on child autistic traits (*N* = 40) were excluded, resulting in 179 participants for analyses. Table 1 presents the baseline characteristics of the study sample and Fig. 1 shows a flow chart of participant inclusion.

**Table 1:** Participant characteristics

Characteristics	Generation R cohort *n* = 4659	CUSP cohort *n* = 179
*Maternal*		
Age (years)	31.5 (4.7)	32.6 (4.5)
Autistic traits score—AQ short	50.8 (8.8)	53.3 (11.6)
Body mass index (BMI), pre-pregnancy	23.1 (3.9)	21.7 (3.9)
*Paternal*		
Age (years)	33.9 (5.4)	35.4 (6.2)
*Child*		
Gestational age (weeks)	39.8 (1.8)	39.5 (1.5)
Sex at birth (%)		
Male	49.4	48.6
Female	50.6	51.4
Birth weight (grams)	3427.6 (580.4)	3402.8 (509.8)
Apgar score at 5 min	9.6 (0.8)	9.7 (0.7)
Autistic traits score^a^	4.9 (3.9)	30.1 (8.2)

Data represent means (SDs) unless specified otherwise

^a^Autistic traits were measured by SRS in The Generation R, by Q-CHAT in CUSP cohort

^*^p < .05 for comparison between The Generation R versus CUSP cohort

### Measures

*Maternal autistic traits* To obtain self-reports of autistic traits in adults, the Generation R and the CUSP cohort used the Autism Spectrum Quotient (AQ)—Adult version (10). In the Generation R, AQ in the mothers was measured when the children were 9 years of age while in the CUSP cohort AQ was measured during pregnancy or shortly after. The Generation R used the validated 28-item abbreviated version of the Autism Spectrum Quotient (AQ-short) (28). In order to compare AQ scores across cohorts, we also calculated the abbreviated 28-item scores for the CUSP cohort. The AQ-short comprises descriptive statements assessing personal preferences and habits (e.g., ‘*My attention is often drawn to car number plates, or similar sequences*’), to which participants responded on a 4-point Likert scale from ‘1 = *definitely agree*’ to ‘4 = *definitely disagree*’. In the CUSP cohort, the Pearson’s correlation between the abbreviated version and full version sum scores was *r* = 0.93 (*p* < 0.001).

*Child autistic traits* In the Generation R cohort, when children were 5–6 and 13–15 years of age, the 18-item abbreviated form of the Social Responsiveness Scale (SRS) was administered to obtain a quantitative measure of autistic traits (29). With the SRS, parents (in most cases the mother), rated the child’s behaviour over the past six months. The questionnaire comprises descriptive statements assessing child habits *(e.g., ‘My child has repetitive, odd behaviours such as hand flapping or rocking’)*, to which parents responded on a 4-point Likert scale, with possible choices from ‘1 = *not true*’ to ‘4 = *almost always true*’*.* The questionnaire was mostly completed by the mothers (participation rate: 52.5% at age 5–6 years and 47.6% at age 13–15 years). The SRS short form, as used in the Generation R sample, is highly correlated (*r* = 0.95) with the full 65 item version (30–32). Internal consistency was estimated for males and females separately (Cronbach’s α males and females: 0.92).

In the CUSP cohort, questionnaires were sent to the mothers, as fathers and other primary caregivers were not formally enrolled in the study. Mothers were invited via email to complete the 25-item online version of the Quantitative Checklist for Autism in Toddlers (Q-CHAT) (33) after their infant reached 18 months of age. The Q-CHAT has been used and validated in the general populations (34, 35). Q-CHAT items describe toddlers’ behaviours and interests, and each item is scored on a 5-point Likert scale measuring frequency, for example: *‘Does your child do the same thing over and over again (e.g., running the tap, turning the light switch on and off, opening and closing doors)’* with possible answering options: ‘A = *many times a day*; ‘B = *a few times a day*; ‘C = *a few times a week*; ‘D = *less than once a week*’ and ‘E = *never*’.

*Maternal Pre-pregnancy BMI* In the Generation R cohort, information about maternal height and weight before pregnancy was obtained by questionnaire. While maternal height and weight during the first trimester of pregnancy were objectively measured at the research centre visit, we used the self-reported measure given our focus on pre-pregnancy BMI. The correlation between self-reported pre-pregnancy weight and weight measured at the enrolment visit was 0.95 (*p* < 0.001). In the CUSP cohort, information about maternal weight and height before pregnancy was obtained by questionnaire based on memory recall of their weight and height at 18 years of age. In addition, CUSP also measured maternal weight and height during first weeks of pregnancy in the first ultrasound appointment. The correlation of self-reported pre-pregnancy weight and height at age 18 years and weight and height measured at the first ultrasound appointment was 0.67 (*p* < 0.001). BMI was calculated as weight in kilograms divided by the square of height in metres (kg/m^2^) for both cohorts.

### Covariates

Several variables (maternal age, paternal age, maternal ethnicity, delivery mode, parity, child sex, birthweight, gestational age, and Apgar score at 5 min) were considered as possible covariates based on previous studies. Covariates were only added into the model if they changed the main effect estimates by more than 5%. Consequently, only maternal age, paternal age, child sex, birthweight, gestational age and Apgar score at 5 min were included in the main analyses.

Information on the age of the parents in both cohorts was obtained at the time of recruitment into the study. Information on sex, birth weight, gestational age, and Apgar score of the child in both cohorts was obtained from medical records completed by community midwives and obstetricians at birth.

### Statistical analyses

In this study, we employed multiple linear regression analyses. First, we examined the association between maternal autistic traits and child autistic traits, using two models. The first model was unadjusted. In the second model, covariates were added. Second, we examined the association between maternal pre-pregnancy BMI and child autistic traits, applying the same two models. For each analysis, we checked for potential nonlinearity of associations by introducing quadratic terms into the model. Next, we examined the interaction between maternal autistic traits and maternal pre-pregnancy BMI in relation with child autistic traits. In addition, we plotted the interaction by using the *linearitycheck* argument in R to see whether estimated lines fit with the predicted lines. All variables were standardized into z-scores for ease of comparison across various time points, resulting in standardized regression coefficients. We also calculated the Cohen’s ƒ^2^ using *R*^*2*^ from the multiple regression output.

For missing data on maternal autistic traits and covariates, multiple imputation was used. Twenty imputed datasets were generated, and pooled estimates were calculated. The variables with missing values in the Generation R cohort were maternal autistic traits (31.4%), pre-pregnancy BMI (23.7%), paternal age (12.3%), Apgar score (7.4%), gestational age (0.6%) and birth weight (0.2%). These data were missing completely at random as tested by Little’s MCAR test (χ^2^ (153) = 686.2, *p* = 0.07). In the CUSP cohort, the variables with missing values were maternal autistic traits (9.5%), pre-pregnancy BMI (14.0%), paternal age (6.7%), Apgar score (14.5%), gestational age (12.8%) and birth weight (12.3%). These data were also missing completely at random as tested by Little’s MCAR test (χ^2^ (71) = 90.1, *p* = 0.06). Additionally, we calculated a priori how many samples were needed with seven variables in the regression model and assuming a moderate effect size of Cohen’s *d* = 0.50 by using G*Power (36). The analysis indicated a minimum sample size of *n* = 150 children, suggesting that our study samples were large enough to detect moderate effect sizes of maternal autistic traits and pre-pregnancy BMI. We analysed the data for both cohorts separately.

### Sensitivity analyses

In CUSP cohort, we re-ran the analysis by using AQ scores calculated based on all items of the questionnaire, rather than the validated short version. We also re-ran the analysis by using maternal BMI measured at the first ultrasound, rather than the maternal BMI at 18 years, to determine the role of the potential large time gap between BMI at 18 years and the pregnancy. For the Generation R, we re-ran the analysis by using the child autistic traits score at 5–6 years of age to check whether findings were consistent across ages. In addition, we re-ran the analyses in the complete cases only. Further, in the Generation R cohort, maternal glycaemic index measured at intake during early pregnancy was examined as a possible moderator. Previous studies have shown a positive association between maternal glycaemic index and maternal BMI (37–39), with glycaemic index potentially better reflecting certain dietary patterns and food choices than body weight. See Wiertsema et al. [40] for details of how glycaemic index was measured in the Generation R cohort. Finally, for clinical relevance and to reveal any potential U-shaped relations, we ran a sensitivity analysis by categorizing maternal pre-pregnancy BMI into four groups corresponding to recommendations of the World Health Organization and previous studies (41–43). The primary comparison contrasted maternal pre-pregnancy BMI groups: comparing healthy BMI (18.5–24.9 kg/m^2^) with underweight (≤ 18.5 kg/m^2^), overweight (25.0–29.9 kg/m^2^) and obesity (≥ 30.0 kg/m^2^) by using ANCOVA.

## Results

### Sample characteristics

Child and maternal characteristics are presented in Table 1. Maternal mean age at enrolment was 31.5 years (SD = 4.7 years) in the Generation R cohort and 32.6 years (4.5 years) in the CUSP cohort. Maternal autistic traits mean scores were higher in the CUSP cohort (*M* = 53.3, SD = 11.6) than in the Generation R cohort (*M* = 50.8, SD = 8.8); however, the effect size for the mean difference was relatively small (*d* = 0.03). Of the children, 49.4% were boys in the Generation R cohort, and 48.6% in the CUSP cohort. Maternal and paternal age correlated strongly (*r* = 0.61 in both cohorts). The correlations between all study variables are presented in Table 2.

**Table 2:** Pearson correlations of variables in the models

	CUSP cohort (*n* = *179*)								
	1	2	3	4	5	6	7	8	9
1. Maternal age		− .112	** − .253**	.**615**	.104	.091	**.197**	− .049	− .125
2. Maternal autistic traits	** − .094**		− .062	− .110	.023	− .046	− .052	.075	**.263**
3. Maternal BMI	− .020	**.053**		− **.198**	− .077	.127	− .048	− .130	.008
4. Paternal age	**.617**	− .036	− .007		− .096	− .013	.076	− .016	.009
5. Gestational age	.006	− .017	− .001	.**036**		− .009	**.616**	.054	− .116
6. Gender (0 = boy; 1 = girl)	− .010	.005	.003	.009	− .012		.110	.074	.057
7. Birth weight	**.067**	** − .048**	**.084**	**.066**	**.637**	** − .118**		.137	− .028
8. Apgar score	.009	− .007	** − .063**	**.056**	**.044**	**.049**	.**044**		.099
9. Child autistic traits	** − .081**	**.166**	**.047**	** − .064**	− .021	** − .100**	** − .038**	− .021	
	Generation R cohort (*n* = *4,659*)								

Bold denotes statistically significant (*p* < .05)

We conducted a non-response analysis to examine any potential patterns or differences between participants who responded to the survey and those who did not. In the CUSP cohort, there were 40 participants excluded and no significant differences were found on several characteristics compared to those who were included in the study, see details in Additional file 1: Table S1. In the Generation R, those who were excluded were younger (*M* = 29.1 years, SD = 5.4, *F* (1, 2047) = 322.9, *p* =  < 0.01), had a higher pre-pregnancy BMI (*M* = 24.0, SD = 4.6, *F* (1, 1461) = 24.4, *p* =  < 0.01), and higher maternal autistic traits (*M* = 52.7 SD = 9.2, *F* (1, 482) = 18.3, *p* =  < 0.01), see details in Additional file 1: Table S1.

### Association between maternal autistic traits, maternal BMI and child autistic traits

The results of the regression analyses on the association of maternal autistic traits and child autistic traits are shown in Table 3. Higher maternal autistic traits were associated with higher child autistic traits at 13 years (Generation R; *β*_*model1*_ = 0.17, *p* < 0.01, ƒ^2^ = 0.04) and at 18 months (CUSP; *β*_*model1*_ = 0.21, *p* < 0.01, ƒ^2^ = 0.05). This association remained statistically significant in the fully adjusted model both in the Generation R (*β*_*adjusted*_ = 0.16, *p* < 0.01, ƒ^2^ = 0.04) and the CUSP cohort (*β*_*adjusted*_ = 0.20, *p* < 0.01, ƒ^2^ = 0.05).).

**Table 3:** Association between maternal autistic traits and child autistic traits

	*β* for Child Autistic Traits (95% *CI)*			
	Generation R		CUSP	
*Model 1*^*a*^				
Maternal Autistic traits	**.17**	**( .13; .19)**	**.21**	**( .06; .35)**
*Model 2*^*b*^				
Maternal Autistic traits	**.16**	**( .13; .19)**	**.20**	**( .06; .36)**
Maternal age	− .03	( − .08; .01)	** − .22**	**( − .41; − .03)**
Paternal age	− .03	( − .07; .02)	**.19**	**( .00; .38)**
Child sex	** − .10**	**( − .14; − .07)**	.07	( − .08; .22)
Birth weight	− .01	( − .06; .03)	.03	( − .16; .22)
Gestational age	− .01	( − .05; .04)	− .12	( − .30; .06)
Apgar score	− .00	( − .04; .03)	.05	( − .09; .20)

^a^ Unadjusted Model

^b^ Adjusted model with confounders: maternal age, paternal age, child sex, birthweight, gestational age

and Apgar score at 5 min

*β* = standardized beta, 95% *CI* = 95% confidence interval

Bold denotes statistical significance (*p* < .05)

Table 4 shows that the association of maternal pre-pregnancy BMI and child autistic traits was statistically significant in the Generation R cohort but not in the CUSP cohort (CUSP: *β*_*adjusted*_ =—0.02, *p* = 0.655). Higher maternal pre-pregnancy BMI was associated with higher child autistic traits at 13 years (Generation R: *β*_*adjusted*_ = 0.03, *p* < 0.01), see details in Table 4. This relationship was linear (test of nonlinearity *p* = 0.18), see details in Additional file 1: Figure S1.

**Table 4:** Association between maternal pre-pregnancy BMI and child autistic traits

	*β* for child autistic traits (95% *CI)*			
	Generation R		CUSP	
*Model 1*^*a*^				
Maternal pre-pregnancy BMI	**.03**	**( .01; .07)**	-.02	( − .19; .14)
*Model 2*^*b*^				
Maternal pre-pregnancy BMI	**.03**	**( .01; .08)**	-.02	( − .23; .13)
Maternal age	** − .06**	**( − .10; − .02)**	-.18	( − .39; .03)
Paternal age	− .03	( − .07; .01)	.16	( − .07; .39)
Child sex	** − .11**	**( − .14; − .08)**	.08	( − .09; .25)
Birth weight	− **.06**	**( − .10; .02)**	.01	( − .21; .22)
Gestational age	.02	( − .02; .05)	-.04	( − .25; .17)
Apgar score	− .01	( − .03; .02)	.07	( − .09; .23)

^a^Unadjusted Model

^b^Adjusted model with confounders: maternal age, paternal age, child sex, birthweight, gestational age and Apgar score at 5 min

*β* = standardized beta, 95% *CI* = 95% confidence interval

Bold denotes statistical significance (*p* < .05)

Table 5 shows the investigation of whether the associations among maternal autistic traits and child autistic traits were moderated by maternal pre-pregnancy BMI. The interaction terms between maternal autistic traits and maternal BMI were not significant in either the Generation R cohort (*β*_*adjusted*_ =—0.01, *p* = 0.734) or the CUSP cohort (*β*_*adjusted*_ = − 0.12, *p* = 0.443).

**Table 5:** Interaction between maternal autistic traits and maternal pre-pregnancy BMI in relation with child autistic traits

	*β* for child autistic traits (95% *CI)*			
	Generation R		CUSP	
Maternal Autistic Traits	**.16**	**(.12; .19)**	**.20**	**(.07; .36)**
Maternal Pre-pregnancy BMI	.03	( − .00; .07)	− .02	( − .19; .15)
Interaction between Maternal Autistic Traits and Maternal Pre-pregnancy BMI	− .01	( − .04; .03)	− .12	( − .27; .02)

*β* = standardized beta, 95% *CI* = 95% confidence interval

Bold denotes statistical significance (*p* < .05)

### Sensitivity analyses

Sensitivity analysis in Generation R on cases with complete information on all variables (*N* = 2202) showed a similar association between maternal autistic traits and child autistic traits (*β*_*adjusted*_ = 0.20, *p* < 0.01), see Additional file 1: Table S2. Sensitivity analyses in the CUSP cohort, using the full AQ version instead of the abbreviated version, also showed similar patterns for the association of maternal autistic traits with child autistic traits, see Additional file 1: Table S3. In addition, we ran the analyses using maternal BMI at the first ultrasound visit instead of at age 18 and it showed similar results for the association between maternal BMI at the first ultrasound and child autistic traits (*β*_*adjusted*_ = -0.01, *p* = 0.334), see Additional file 1: Table S4. Sensitivity analyses in the Generation R cohort using child autistic traits score at 5–6 years of age (instead of at 13 years) also showed similar results for the associations between maternal autistic traits and child autistic traits (*β*_*adjusted*_ = 0.19, *p* < 0.01, ƒ^2^ = 0.04, Additional file 1: Table S5), and between maternal pre-pregnancy BMI and child autistic traits (*β*_*adjusted*_ = 0.04, *p* < 0.01, Additional file 1: Table S6). In addition, maternal glycaemic index also did not moderate the association between maternal autistic traits and child autistic traits (data not shown). In none of the sensitivity analyses, the association between maternal autistic traits and child autistic traits was moderated by maternal pre-pregnancy BMI.

In our categorical approach, we found a statistically significant mean difference between the maternal pre-pregnancy BMI groups in the Generation R cohort as determined by a one-way ANCOVA, (F_3, 4657_ = 9,571, *p* < 0.01) (see Table 6). A Tukey post hoc test revealed that child autistic traits scores were significantly higher in mothers who were underweight (0.23 ± 0.08, *p* < 0.01) and in mothers who were overweight (0.17 ± 0.03, *p* < 0.01) compared to the mothers with a healthy weight. There was no statistically significant difference in child autistic traits scores between the healthy weight and obesity groups (*p* = 0.09). In the CUSP cohort, we did not find any statistically significant differences between the maternal pre-pregnancy BMI groups in child autistic traits scores.

**Table 6:** Relation between maternal pre-pregnancy BMI groups and child autistic traits scores

Maternal pre-pregnancy BMI groups		Mean (SD)	Sig	*d*
Generation R *(N* = *4,659)*				
Underweight (≤ 18.5)	135	5.7 (5.2)	.042	0.3
* Healthy (18.5–24.9)*	*3401*	*4.7 (3.7)*	*–*	*–*
Overweight (25.0–29.9)	879	5.4 (4.6)	.000	0.2
Obesity (≥ 30.0)	244	5.3 (3.6)	.099	*–*
**CUSP (*N*** **=** ***179)***				
Underweight (≤ 18.5)	22	33.1 (8.3)	.274	*–*
* Healthy (18.5–24.9)*	*134*	*29.7 (8.2)*	*–*	*–*
Overweight (25.0–29.9)	18	27.1 (5.5)	.664	*–*
Obesity (≥ 30.0)	5	36.4 (10.1)	.129	*–*

Confounders that were included were maternal age, paternal age, child sex, birthweight, gestational age and Apgar score at 5 min

## Discussion

In two independent cohorts, we found that maternal autistic traits were positively associated with autistic traits in their offspring throughout childhood, albeit with relatively small effect sizes. In Generation R cohort, maternal pre-pregnancy BMI was found to be independently, positively and linearly associated with child autistic traits; however, in both cohorts, there was no interaction of maternal pre-pregnancy BMI on the association between autistic traits of mothers and their children. The finding that higher autistic traits in mothers were associated with higher autistic traits in children is consistent with previous studies (5, 44). The effect size we found in this study is comparable (ƒ^2^_*toddlerhood*_ = 0.05; ƒ^2^_*earlychildhood*_ = 0.04; ƒ^2^_*earlyadolescence*_ = 0.04) to Kröger et al. [5] study (ƒ^2^ = 0.04). In addition, our finding in the Generation R cohort that maternal pre-pregnancy BMI was positively associated with child autistic traits both in middle childhood and adolescence has only been shown a few times before (12, 14). This association may be explained by other clinical factors associated with a higher BMI in pregnancy. For example, excessive BMI is a significant risk factor for maternal diabetes, which in itself also significantly increases the likelihood of autism in the offspring (45). In addition, this result might be explained by previous work in the Generation R cohort that reported that a higher maternal pre-pregnancy BMI was associated with advanced foetal growth (46) and that mothers with the highest pre-pregnancy BMI had a higher likelihood of delivering infants with a large size for gestational age (47). In addition, being large for gestational age was previously reported to be a likelihood factor for autistic traits later in life (48). However, this likelihood was small, and biological explanations remain to be investigated. This line of reasoning might also explain the discrepancy in findings between the Generation R cohort and the CUSP cohort, where no significant association was found for maternal pre-pregnancy BMI and child autistic traits: in the CUSP cohort, pregnancies with large foetal size for gestational age were excluded from the study.

Another reason for not finding a significant association between maternal pre-pregnancy BMI and child autistic traits in the CUSP cohort might lie in the assessment of BMI, which was based on memory recall back to when mothers were 18 years of age. Generally, memory recall seems less accurate if more time has elapsed (49), while studies also reported that woman tend to underestimate their past weight (50, 51). Therefore, we adjusted the analyses for maternal age during pregnancy, which is indicative of the years of memory recall that we relied on, this recall bias might have affected our results. In contrast to our findings, previous studies were able to detect the association between maternal pre-pregnancy BMI and child autistic traits even though pre-pregnancy BMI was based on recall memory of their weight and height at 18 years of age (19, 52). Importantly, a study by Getz et al. [15] on the association of maternal BMI and child autistic traits revealed that associations were generally stronger for BMI measurements obtained closer to the start of the pregnancy. However, in our study, a similar null-finding was obtained when we performed a sensitivity analysis with maternal BMI at first ultrasound.

In both cohorts, we did not find an interaction effect between maternal pre-pregnancy BMI and maternal autistic traits in the association with child autistic traits, even though our power analyses indicated that our sample sizes were large enough to detect differences. The lack of interaction may be due to little variation in maternal pre-pregnancy BMI in both cohorts. In both cohorts the BMI distribution concentrated around mothers with a healthy BMI, while fewer mothers than expected based on population indices were overweight or obese (47). We recommend future studies to include larger sample sizes or to oversample respondents who are overweight or obese. Nevertheless, it is also important to acknowledge the possibility that maternal BMI may not serve as a moderator in the association between maternal and child autistic traits, suggesting that maternal BMI might not play a substantial role in influencing the relationship between these traits. As observed in the analyses with maternal autistic traits included in the model, the link between maternal BMI and child autistic traits diminishes and is no longer statistically significant. Along these lines, a study conducted by Gardner et al. [53] showed that maternal BMI and the likelihood of autism were not associated, particularly when considering unmeasured familial influences through sibling analyses. Furthermore, it may be that the association between maternal BMI and child autistic traits does not follow a linear pattern across the entire range, but rather more evident categorically in the extremes of the distributions. For instance, this might be particularly true for an autism diagnosis and obesity (19), rather than for milder autistic traits or overweight.

### Methodological considerations

It is important to interpret our findings in light of some methodological considerations. First, our study is limited by the difference in temporal timeline of weight and height measures between Generation R and CUSP cohort. Comparing the weight and height information immediately before pregnancy from Generation R, with the CUSP cohort in which BMI at 18 years of age was measured instead, could lead to discrepancies due to the considerable time gap between the two assessments (i.e., fluctuating weight over past years). However, a sensitivity analysis with maternal pre-pregnancy BMI data from the initial ultrasound in CUSP resulted in similar results.

Second, it is important to highlight that there was a significant amount of missing data in the Generation R cohort and the non-response analysis revealed that those with a higher BMI and more autistic traits were more likely to be non-responders. This may have led to an underestimation of the associations. Another concern in this study is the presence of unmeasured confounding, primarily attributed to genetics. Notably, a study (54) employing phenotypic and genotypic data from 2614 trios has reported that elevated polygenic scores for autism are associated with autistic traits in mothers of children with autism. The findings of this study (54) underscore the importance of considering multiple confounding factors, both at the phenotypic and genotypic level, in understanding the association between parental and child autistic traits and the moderating factors influencing this association. We recommend future studies to addressed this confounding by adjusting for polygenic scores (55).

Finally, sensitivity analysis using a categorical approach of maternal pre-pregnancy BMI showed that in the Generation R cohort, not only a higher maternal weight (overweight), but also a lower maternal weight (underweight) was associated with higher autistic traits scores in childhood and adolescence. However, in the CUSP cohort, findings for the underweight and higher maternal weight groups were in a similar direction, though not statistically significant. Potentially, the analyses with weight groups might have had limited power to detect differences, particularly due to low numbers in the underweight and obesity categories.

## Conclusion

Using two different cohort datasets from the UK and the Netherlands enabled us to uncover whether maternal autistic traits and weight are related with child autistic traits in different developmental periods (i.e., toddlerhood, early childhood, and early adolescence). In conclusion, we showed that in the Generation R cohort, both maternal autistic traits and maternal pre-pregnancy BMI were positively associated with child autistic traits in early childhood and early adolescence, yet with small effect sizes (ƒ^2^ = 0.04). Results from the analyses with weight categories are indicative of a nonlinear link with the more extreme ends of the maternal pre-pregnancy BMI spectrum being associated with more child autistic traits. In CUSP cohort, maternal autistic traits, but not maternal BMI, were positively associated with child autistic traits in toddlerhood, with a small effect size (ƒ^2^ = 0.05). We did not find an interaction of maternal pre-pregnancy BMI on the association between autistic traits of mothers and their children in both Generation R and CUSP cohort.

Future research is warranted to examine our tentative findings further. Such investigation should, however, be done in a large enough sample with sufficient numbers of women included at the lower as well as higher end of the BMI distribution. Additional studies that model pre-pregnancy BMI along with additional characteristics (e.g., maternal diet during pregnancy, maternal immune activation, polygenic score) may be needed to further understand the mechanisms underlying the role of maternal weight on the development of child autistic traits. In particular, the scope of this study may be extended to examining additional independent variables, such as prenatal inflammation, placental function and maternal mental health during and after pregnancy.

### Supplementary Information

**Additional file 1.** Supplementary figure and tables.

## Data Availability

The datasets analysed during the current study are not publicly available due to the terms and conditions participants agree to when they participate in the study, but are available from the corresponding author on reasonable request.
