# Supplementary material for: Is the association between mothers’ autistic traits and childhood autistic traits moderated by maternal pre-pregnancy body mass index?
Source: Mol Autism. 2023 Dec 8;14:46. doi: 10.1186/s13229-023-00578-x (PMC10709910; doi:10.1186/s13229-023-00578-x)
Supplement: Supplementary file 1 — Additional file 1. Supplementary figure and tables. [file 13229_2023_578_MOESM1_ESM.docx]

**Supplementary Figure 1**

*Interaction plot of maternal pre-pregnancy BMI in the association between maternal and child autistic traits with non-linear predicted lines.*

**
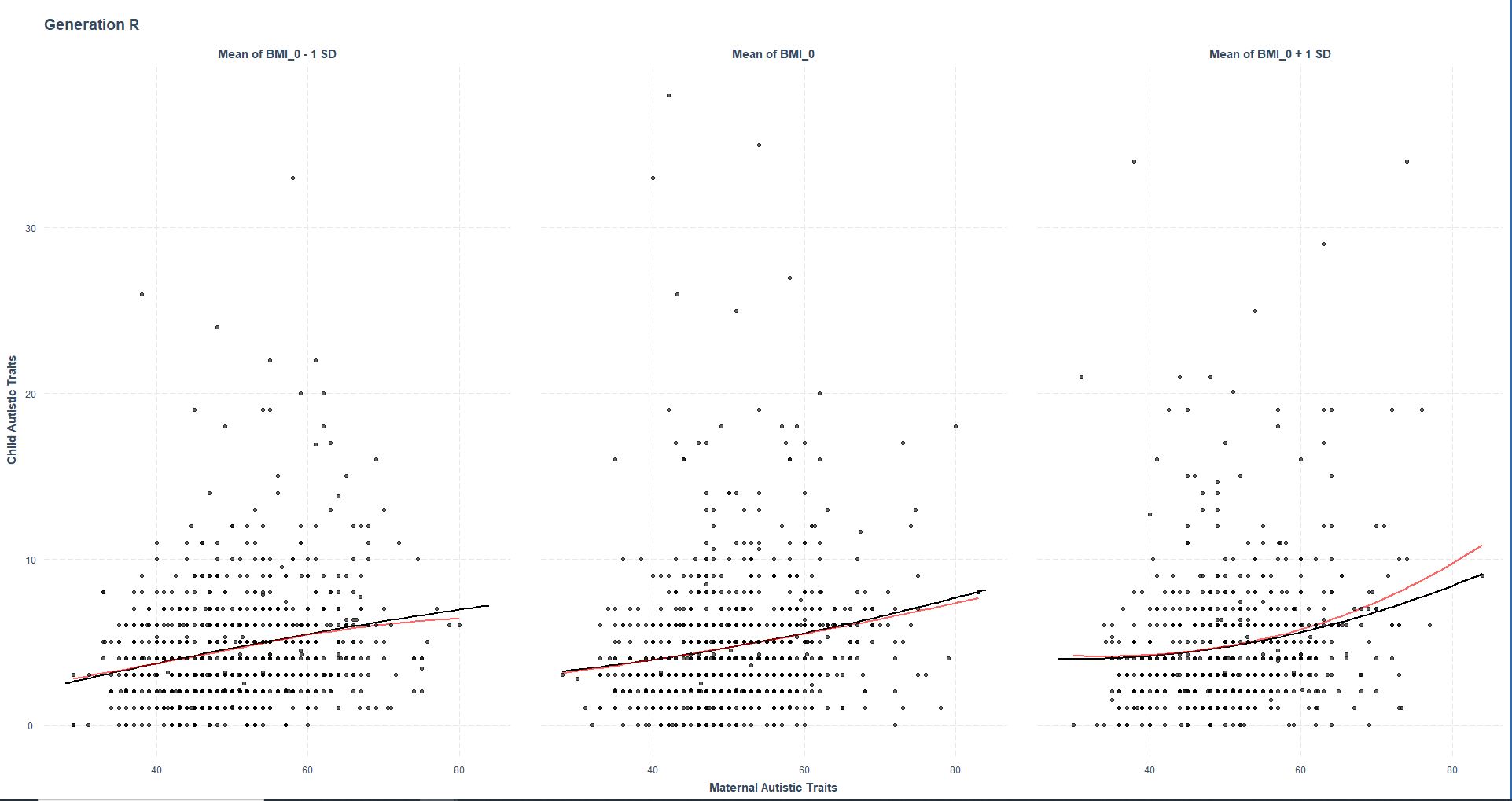
**

**
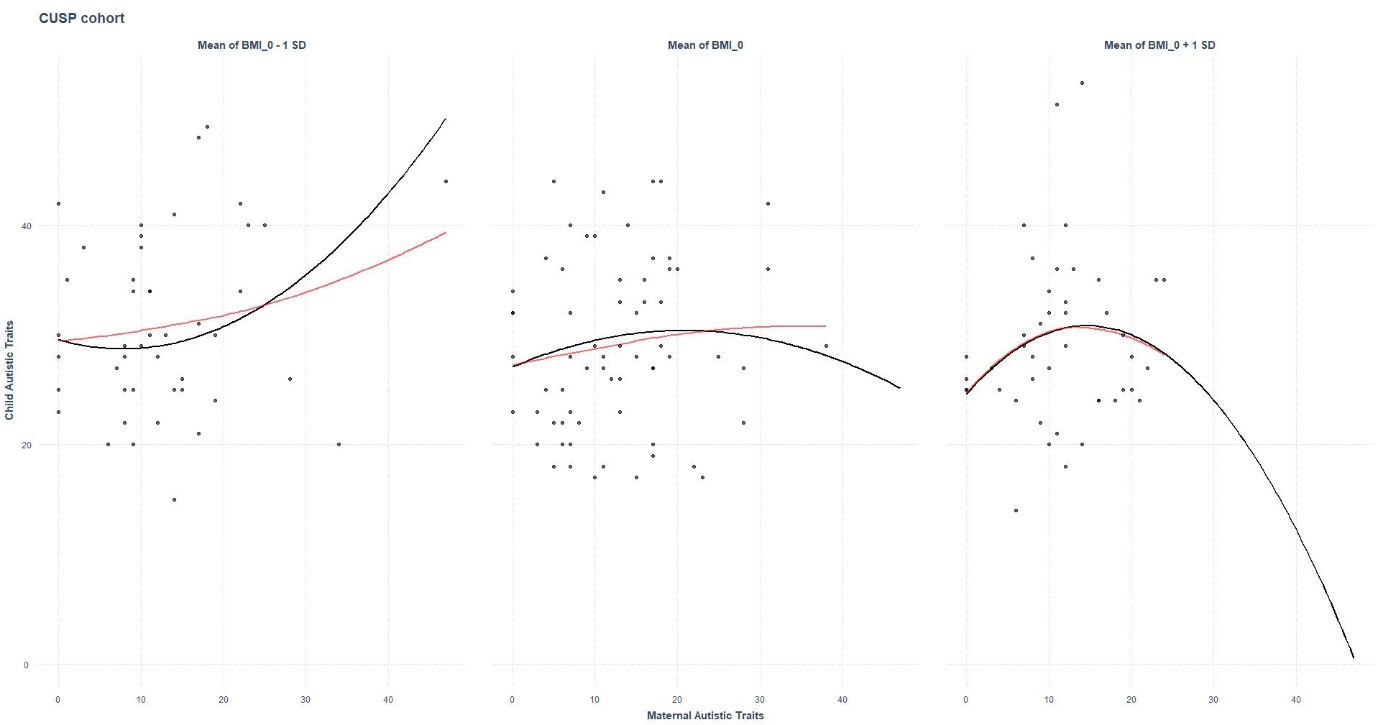
**

Note. Black line represents non-linear predicted lines coming from the full data set and red line represents data at the subset of data in each level and will be curved if the relationship is non-linear.

**Supplementary Table 1**

*Sensitivity Analysis on Participant Characteristics Included vs Excluded from the Study*

| **Generation R** | **Included**  ***n* = 4,659** | **Excluded**  ***n* = 2047** |
| --- | --- | --- |
| *Maternal* |  |  |
| Age (years) | 31.5 (4.7) | 29.1 (5.4)* |
| Autistic Traits score – AQ short | 50.8 (8.8) | 52.7 (9.2)* |
| Body Mass Index (BMI),  pre-pregnancy | 23.1 (3.9) | 24.0 (4.6)* |
| *Paternal* |  |  |
| Age (years) | 33.9 (5.4) | 32.5 (6.2)* |
|  |  |  |
| *Child* |  |  |
| Gestational Age (weeks) | 39.8 (1.8) | 39.7 (1.8) |
| Sex at Birth (%) |  |  |
| Male | 49.4 | 51.9 |
| Female | 50.6 | 48.1 |
| Birth Weight (grams) | 3427.6 (580.4) | 3366.3 (563.1)* |
| Apgar score at 5 minutes | 9.6 (0.8) | 9.6 (0.8) |
| Autistic Traits score^a^ | 4.9 (3.9) | - |
|  |  |  |

| **CUSP** | **Included**  ***n* = 179** | **Excluded**  ***n* = 40** |
| --- | --- | --- |
| *Maternal* |  |  |
| Age (years) | 32.6 (4.5) | 31.5 (4.5) |
| Autistic Traits score – AQ short | 53.3 (11.6) | 53.2 (11.4) |
| Body Mass Index (BMI),  pre-pregnancy | 21.7 (3.9) | 20.7 (3.5) |
| *Paternal* |  |  |
| Age (years) | 35.4 (6.2) | 32.8 (6.1) |
|  |  |  |
| *Child* |  |  |
| Gestational Age (weeks) | 39.5 (1.5) | 39.6 (1.7) |
| Sex at Birth (%) |  |  |
| Male | 48.6 | 48.6 |
| Female | 51.4 | 51.4 |
| Birth Weight (grams) | 3402.8 (509.8) | 3445.2 (534.7) |
| Apgar score at 5 minutes | 9.7 (0.7) | 9.8 (0.6) |
| Autistic Traits score^a^ | 30.1 (8.2) | - |
|  |  |  |

*Note.* Data represent means (SDs) unless specified otherwise.

*p < .05 for comparison between participants who are included versus excluded from the cohort.

**Supplementary Table 2**

*Sensitivity Analysis in Generation R using Complete Cases (N = 2202)*

|  | *β* for Child Autistic Traits (95% *CI)* | |
| --- | --- | --- |
|  |  | |
| *Model 1^a^* |  |  |
| Maternal Autistic Traits | **.20** | **( .15 ; .23 )** |
| *Model 2^b^* |  |  |
| Maternal Autistic Traits | **.20** | **( .15 ; .23 )** |
| Maternal Age | -.03 | ( -.09 ; .03 ) |
| Paternal Age | -.01 | ( -.01 ; .05 ) |
| Child Sex | **-.10** | **( -.14 ; -.06 )** |
| Birth Weight | -.01 | ( -.06 ; .05 ) |
| Gestational Age | -.06 | ( -.11 ; .00 ) |
| Apgar Score | -.01 | ( -.05 ; .03 ) |

^a^ Unadjusted Model

^b^ Adjusted model with confounders: maternal age, paternal age, child sex, birthweight, gestational age

and Apgar score at 5 minutes.

*β* = standardized beta, 95% *CI* = 95% confidence interval.

Bold denotes statistical significance (*p* < .05).

**Supplementary Table 3**

*Sensitivity Analysis in the CUSP Cohort using the Full AQ instead of the AQ-short*

|  | *β* for Child Autistic Traits (95% *CI)* |
| --- | --- |

| *Model 1^a^* |  |  |  |
| --- | --- | --- | --- |
| Maternal Autistic Traits* |  | **.21** | **( .06 ; .35 )** |
| *Model 2^b^* |  |  |  |
| Maternal Autistic Traits* |  | **.20** | **( .06 ; .36 )** |
| Maternal Age |  | **-.22** | **( -.41 ; -.03 )** |
| Paternal Age |  | **.19** | **( .00 ; .38 )** |
| Child Sex |  | .07 | ( -.08 ; .22 ) |
| Birth Weight |  | .03 | ( -.16 ; .22 ) |
| Gestational Age |  | -.12 | ( -.30 ; .06 ) |
| Apgar Score |  | .05 | ( -.09 ; .20 ) |

^a^ Unadjusted Model

^b^ Adjusted model with confounders: maternal age, paternal age, child sex, birthweight, gestational age

and Apgar score at 5 minutes.

*β* = standardized beta, 95% *CI* = 95% confidence interval.

Bold denotes statistical significance (*p* < .05).

**Supplementary Table 4**

*Sensitivity Analysis in the CUSP Cohort using Maternal Pre-pregnancy BMI at*

*First Ultrasound.*

|  | *β* for Child Autistic Traits (95% *CI)* | |
| --- | --- | --- |
|  |  | |
| *Model 1^a^* |  |  |
| Maternal Pre-pregnancy BMI | -.01 | ( -.17 ; .11 ) |
| *Model 2^b^* |  |  |
| Maternal Pre-pregnancy BMI | -.01 | ( -.23 ; .13 ) |
| Maternal Age | -.15 | ( -.39 ; .03 ) |
| Paternal Age | .12 | ( -.07 ; .39 ) |
| Child Sex | .07 | ( -.09 ; .25 ) |
| Birth Weight | .01 | ( -.21 ; .22 ) |
| Gestational Age | -.03 | ( -.25 ; .17 ) |
| Apgar Score | .07 | ( -.09 ; .23 ) |

^a^ Unadjusted Model

^b^ Adjusted model with confounders: maternal age, paternal age, child sex, birthweight, gestational age

and Apgar score at 5 minutes.

*β* = standardized beta, 95% *CI* = 95% confidence interval.

*Sensitivity analysis in CUSP cohort using maternal BMI at the first ultrasound.

Bold denotes statistical significance (*p* < .05).

**Supplementary Table 5**

*Sensitivity Analysis in Generation R on the Association between Maternal*

*Autistic Traits and Child Autistic Traits at age 5-6 years*

|  | *β* for Child Autistic Traits (95% *CI)* | |  |
| --- | --- | --- | --- |
| *Model 1^a^* |  |  |  |
| Maternal Autistic Traits* | **.21** | **( .17 ; .24 )** |  |
| *Model 2^b^* |  |  |  |
| Maternal Autistic Traits* | **.19** | **( .16 ; .23 )** |  |
| Maternal Age | **-.07** | **( -.12 ; -.02 )** |  |
| Paternal Age | -.01 | ( -.04 ; .06 ) |  |
| Child Sex | **-.16** | **( -.19 ; -.13 )** |  |
| Birth Weight | -.02 | ( -.07 ; .03 ) |  |
| Gestational Age | -.03 | ( -.07 ; .02 ) |  |
| Apgar Score | -.01 | ( -.03 ; .04 ) |  |

^a^ Unadjusted Model

^b^ Adjusted model with confounders: maternal age, paternal age, child sex, birthweight, gestational age

and Apgar score at 5 minutes.

*β* = standardized beta, 95% *CI* = 95% confidence interval.

Bold denotes statistical significance (*p* < .05).

**Supplementary Table 6**

*Sensitivity Analysis in Generation R on the Association between Maternal Pre-pregnancy BMI and Child Autistic Traits at 5-6 years*

|  | *β* for Child Autistic Traits  (95% *CI)* | |
| --- | --- | --- |
| *Model 1^a^* |  |  |
| Maternal Pre-pregnancy BMI | **.04** | **( .01 ; .06 )** |
| *Model 2^b^* |  |  |
| Maternal Pre-pregnancy BMI | **.04** | **( .01 ; .07 )** |
| Maternal Age | **-.05** | **( -.10 ; -.02 )** |
| Paternal Age | -.03 | ( -.07 ; .01 ) |
| Child Sex | **-.10** | **( -.14 ; -.08 )** |
| Birth Weight | **-.05** | **( -.10 ; .02 )** |
| Gestational Age | .02 | ( -.02 ; .05 ) |
| Apgar Score | -.01 | ( -.03 ; .02 ) |

^a^ Unadjusted Model

^b^ Adjusted model with confounders: maternal age, paternal age, child sex, birthweight, gestational age

and Apgar score at 5 minutes.

*β* = standardized beta, 95% *CI* = 95% confidence interval.

*Sensitivity analysis using SRS (child autistic traits) measured at 5-6 years in Generation R cohort.

Bold denotes statistical significance (*p* < .05).
